# Endoplasmic reticulum associated degradation preserves neurons viability by maintaining endoplasmic reticulum homeostasis

**DOI:** 10.3389/fnins.2024.1437854

**Published:** 2024-07-29

**Authors:** Shuangchan Wu, Pingting Liu, Marija Cvetanovic, Wensheng Lin

**Affiliations:** ^1^Department of Neuroscience, University of Minnesota, Minneapolis, MN, United States; ^2^Institute for Translational Neuroscience, University of Minnesota, Minneapolis, MN, United States

**Keywords:** ER-associated degradation, ER stress, Purkinje neuron, hippocampal neuron, neurodegeneration

## Abstract

Endoplasmic reticulum-associated degradation (ERAD) is a principal quality-control mechanism responsible for targeting misfolded ER proteins for cytosolic degradation. Evidence suggests that impairment of ERAD contributes to neuron dysfunction and death in neurodegenerative diseases, many of which are characterized by accumulation and aggregation of misfolded proteins. However, the physiological role of ERAD in neurons remains unclear. The Sel1L-Hrd1 complex consisting of the E3 ubiquitin ligase Hrd1 and its adaptor protein Sel1L is the best-characterized ERAD machinery. Herein, we showed that Sel1L deficiency specifically in neurons of adult mice impaired the ERAD activity of the Sel1L-Hrd1 complex and led to disruption of ER homeostasis, ER stress and activation of the unfold protein response (UPR). Adult mice with Sel1L deficiency in neurons exhibited weight loss and severe motor dysfunction, and rapidly succumbed to death. Interestingly, Sel1L deficiency in neurons caused global brain atrophy, particularly cerebellar and hippocampal atrophy, in adult mice. Moreover, we found that cerebellar and hippocampal atrophy in these mice resulted from degeneration of Purkinje neurons and hippocampal neurons, respectively. These findings indicate that ERAD is required for maintaining ER homeostasis and the viability and function of neurons in adults under physiological conditions.

## 1 Introduction

Most secreted and membrane proteins fold and assemble to achieve their functional structures in the endoplasmic reticulum (ER) lumen. Only correctly assembled proteins translocate from the ER to their destination. However, protein folding is susceptible to errors. Unfolded or misfolded proteins are identified, and sent back to the cytosol, where they are degraded by the ubiquitin-proteasome system. This process is known as ER-associated degradation (ERAD) (Nakatsukasa et al., 2014; Sun et al., 2014; Qi et al., 2017; Zhou et al., 2020). Accumulation of misfolded or unfolded proteins in the ER lumen causes ER stress and triggers the unfolded protein response (UPR) (Lin and Popko, 2009; Lin and Stone, 2020; Wu and Lin, 2024). The UPR comprises three parallel branches: pancreatic ER kinase (PERK), inositol requiring enzyme 1 (IRE1), and activating transcription factor 6 (ATF6) (Lin and Popko, 2009; Marciniak et al., 2022; Wiseman et al., 2022; Wu and Lin, 2024). These branches work together to re-establish ER homeostasis by assisting protein folding, attenuating protein translation, and improving ERAD efficiency (Marciniak et al., 2022; Wiseman et al., 2022). The UPR and ERAD are key quality control systems that ensure ER homeostasis.

Evidence suggests that maintaining ER homeostasis is requiring for brain development and memory storage under normal conditions (Godin et al., 2016; Martinez et al., 2018; Wu and Lin, 2024), and that disruption of ER homeostasis contributes to neuronal dysfunction and death in the context of neurodegenerative diseases (Kurtishi et al., 2019; Costa-Mattioli and Walter, 2020; Hetz, 2021). A notable recent study by Liu et al. (2023) demonstrated that impairment of the UPR in neurons, specifically by simultaneous deletion of PERK and ATF6α, causes disruption of ER homeostasis and results in spatial memory impairment and degeneration of the hippocampus in adult mice (Liu et al., 2023). This finding underscores the importance of maintaining ER homeostasis in neuronal function and viability under normal conditions (Liu et al., 2023). Moreover, irregularities in ERAD components, including Derlin, Erlin, Ubiquilin, membralin, and hydroxymethylglutaryl-CoA reductase degradation protein 1 (Hrd1), have been identified as contributing factors to neuronal dysfunction and death in animal model of neurodegenerative diseases (Deng et al., 2011; Omura et al., 2013; Zhu et al., 2017). However, it remains unclear whether and how ERAD participates in regulating neuronal function and viability under normal conditions.

Hrd1, a well-recognized ER-resident E3 ubiquitin ligase crucial for ERAD, forms a complex with its adaptor protein, Suppressor/Enhancer of Lin-12-like (Sel1L) (Gao Y. et al., 2023; Ji et al., 2023; Wang et al., 2024). This Sel1L-Hrd1 complex is responsible for recognition and degradation of certain aberrant proteins in the ER (Gao Y. et al., 2023; Ji et al., 2023; Wang et al., 2024). Our latest research indicates that Sel1L deficiency in oligodendrocytes leads to progressive thinning of myelin in the central nervous system (CNS) of adult mice (Wu et al., 2020; Wu and Lin, 2023). Similarly, Sel1L deficiency in Schwann cells results in delayed apoptosis of these cells and demyelination in the peripheral nervous system (PNS) of adult mice (Wu et al., 2021; Wu and Lin, 2023). This underscores the critical importance of ERAD in the functioning of mature myelinating cells in adults. Therefore, in this study, we aimed to explore the role of ERAD in neurons under normal conditions using mice with inducible Sel1L inactivation specifically in neurons. Interestingly, adult mice with Sel1L inactivation in neurons exhibited weight loss and severe motor dysfunction, and rapidly succumbed to death. Sel1L inactivation in neurons caused global brain atrophy in adult mice, particularly cerebellar and hippocampal atrophy resulting from degeneration of Purkinje neurons and hippocampal neurons. These results suggest that ERAD is indispensable for maintaining ER homeostasis and the viability and function of neurons in the adult CNS.

## 2 Materials and methods

### 2.1 Mice and tamoxifen treatment

We have obtained *Sel1L^loxP^* mice, which possess loxP sites flanking exon 6 of the Sel1L gene, from Dr. Ling Qi (The University of Michigan, Ann Arbor, MI). *Thy1/CreER^T2^* transgenic mice, which express the CreER^T2^ recombinase under the control of the Thy1.2 promoter (Heimer-McGinn and Young, 2011), were purchased from the Jackson Laboratory (stock number 012708). The *Sel1L*^loxP^ mice and *Thy1/CreER^T2^* transgenic mice were on the C57BL/6 J background. *Sel1L^loxP^* mice were crossed with *Thy1/CreER^T2^* mice, and the resulting progeny were further crossed with *Sel1L^loxP^* mice to obtain *Sel1L^loxP/loxP^*; *Thy1/CreER^T2^* mice and *Sel1L^loxP/loxP^* mice. Both male and female mice were used for all the experiments. Genotypes were determined by PCR from DNA extracted from tail tips as described in previous papers (Stone et al., 2019; Wu and Lin, 2023). To induce recombination, *Sel1L^loxP/loxP^; Thy1/CreER^T2^* mice and *Sel1L^loxP/loxP^* mice were given intraperitoneal injections of tamoxifen (Sigma, 50 mg/kg per day) daily for 10 days starting at the age of 8 weeks. To assess Cre-mediated recombination of floxed alleles in neurons of these mice, genomic DNA was isolated from the indicated tissues and PCR was performed as described in previous papers (Wu et al., 2020, 2021; Liu et al., 2023; Wu and Lin, 2023). We monitored mice daily to detect neurological phenotypes. All animal procedures were conducted in complete compliance with the NIH Guide for the Care and Use of Laboratory Animals and were approved by the Institutional Animal Care and Use Committee of the University of Minnesota.

### 2.2 Hindlimb clasping test

Hindlimb clasping test was performed as reported (Guyenet et al., 2010; Liu et al., 2023). There were 8–12 mice in each group. Mice were suspended by the base of the tail and were observed for 10–15 s (s). Three separate trials were taken per day for each mouse. Mice were scored for the severity of hindlimb clasping where 0 indicated normal splaying of hind limbs, 1 indicated transient clasping of one hindlimb, 2 indicated transient clasping of two hindlimbs, 3 indicated severe and sustained hindlimb clasping of one hindlimb, 4 indicated severe and sustained hindlimb clasping of two hindlimbs. A higher score indicates a more severe phenotype.

### 2.3 Accelerating rotarod test

The rotarod apparatus (Panlab/Harvard Apparatus) with a spindle diameter of 3 cm was used to test motor activity of mice (Wu et al., 2020). There were 8–12 mice in each group. For the trials, mice were placed on the rotor rod at 4 rpm, and then the rotor rod was set to accelerate continuously from 4 to 40 rpm over a period of 300 s and the latency to fall was recorded (falls were detected with a pressure sensitive lever); 180 s was the maximum time for the trial, and mice that reached this were recorded as 180 s and removed from the rotarod apparatus.

### 2.4 Western blot

The half brains (bisected in the sagittal plane) harvested from mice were rinsed in ice-cold PBS and were homogenized using a motorized homogenizer as previously described (Liu et al., 2023; Wu and Lin, 2023). There were 4 mice in each group. After incubating on ice for 15 min, the extracts were cleared by centrifugation at 14,000 rpm for 30 min twice. The protein content of each extract was determined by DC Protein Assay (Bio-Rad Laboratories). The extracts (40 mg) were separated by SDS-PAGE and transferred to nitrocellulose membranes. The membranes were incubated with a primary antibody against Sel1L (1:500, Santa Cruz Biotechnology, Cat# sc-377351), Hrd1 (1:2,000, Thermo Fisher Scientific, Cat# PA5-12093, RRID: AB_2199832), or β-actin (1:5,000, Sigma Millipore, Cat# A2103, RRID: AB_476694), followed by an HRP-conjugated secondary antibody (1:1,000, Vector Laboratories anti-mouse, Cat# PI-2000, RRID: AB_2336177; anti-rabbit, Cat# PI-1000, RIDD: AB_2336198). The chemiluminescent signal was detected using the ECL Detection Reagents (GE Healthcare Biosciences). The intensity of the chemiluminescence signals was quantified using the National Institutes of Health ImageJ software. The intensity of individual proteins was normalized to β-actin.

### 2.5 Immunohistochemistry, immunofluorescence, and Nissl staining

Anesthetized mice were perfused through the left cardiac ventricle with 4% PFA in PBS, and the tissues were removed as previously described (Lei et al., 2020; Stone et al., 2020). There were 4 mice in each group. Half sagittal brains were postfixed in 4% PFA for 2 h, cryoprotected in 30% sucrose for 48 h, embedded in optimum cutting temperature compound, and frozen on dry ice. Frozen sections were cut using a cryostat at a thickness of 10 μm. The other half sagittal brains were postfixed in 4% PFA for 72 h, dehydrated through graded alcohols, and embedded in paraffin wax. Paraffin sections were cut using a microtome at a thickness of 5 μm. Immunohistochemical and immunofluorescence detection of NeuN (1:100, Millipore, Cat# MAB337, RRID:AB_2313673), GFAP (1:100; COVΛNCE, LN#14831701), CD11b (1:50; Millipore, Cat# CBL1313, RRID:AB_92930), Calbindin (1:400, Sigma-Aldrich, Cat# C9848, RRID:AB_476894), Sel1L (1:500, Santa Cruz Biotechnology, catalog #sc-377351), CCAAT/enhancer-binding protein homologous protein (CHOP, 1:100, Santa Cruz Biotechnology, Cat# sc-56107, RRID:AB_783507), phosphorylated eIF2α (p-eIF2α, 1:200, Cell Signaling Technology, Danvers, MA, Cat# 9721, RRID:AB_330951), were performed as previously described (Yue et al., 2019; Liu et al., 2023).

Paraffin sections were used for Nissl staining. After deparaffinization, the sections were stained with 0.1% cresyl violet solution for 3 min, rinsed in water, differentiated in 95% alcohol for 3 min, dehydrated in 100% alcohol, and cleared in xylene. Stained brain sections were scanned by a TissueScope LE120 slide scanner (Huron Digital Pathology).

For quantification of Purkinje neuron number and size in the cerebellum, 5 μm thick sagittal brain sections were cut and every tenth sagittal section in the series spanning from Bregma lateral 0.12 mm to 0.36 mm were stained with the calbindin antibody. The total number and size of calbindin-positive neurons in each cerebellum section were analyzed using the NIH ImageJ software. The thickness of the cerebellar molecular layer was quantified using the NIH ImageJ software as described in our previous paper (Hamel et al., 2024). Briefly, we took three measurements per image of the distance from the base of the Purkinje soma to the end of the molecular layer, the average being the thickness of the molecular layer for that image.

For quantification of neurons in the hippocampus, 5 μm thick sagittal brain sections were cut and every tenth sagittal section containing the dorsal hippocampus in the series spanning from Bregma lateral 1.08 mm to 1.56 mm were stained with the NeuN antibody. The total NeuN-positive neurons in the CA1 layer, CA2 layer, CA3 layer, and dentate gyrus (DG) were counted and analyzed using the NIH ImageJ software, as described in our previous paper (Liu et al., 2023).

### 2.6 XBP1 splicing assay

RNA was isolated from the brain using TRIzol reagent (Invitrogen, Thermo Fisher Scientific) following the manufacturer’s instructions, and treated with DNase I (Invitrogen, Thermo Fisher Scientific) to eliminate genomic DNA. Reverse transcription was performed using the iScript cDNA Synthesis Kit (Bio-Rad Laboratories). PCR for XBP1 was performed to detect XBP1s mRNA using Taq DNA Polymerase (QIAGEN). PCR products were assessed by electrophoresis on a 3% agarose gel as previously described (Stone et al., 2018, 2020). There were 3 mice in each group.

### 2.7 Statistical analysis

All data are expressed as mean ± SD (standard derivation). Comparison between two groups was statistically evaluated by *t* test using GraphPad Prism 6 (GraphPad Software, RRID:SCR_002798). *p* values less than 0.05 were considered significant.

## 3 Results

### 3.1 Neuron-specific Sel1L inactivation in adult mice led to weight loss, motor dysfunction, and death

**Figure 1:**
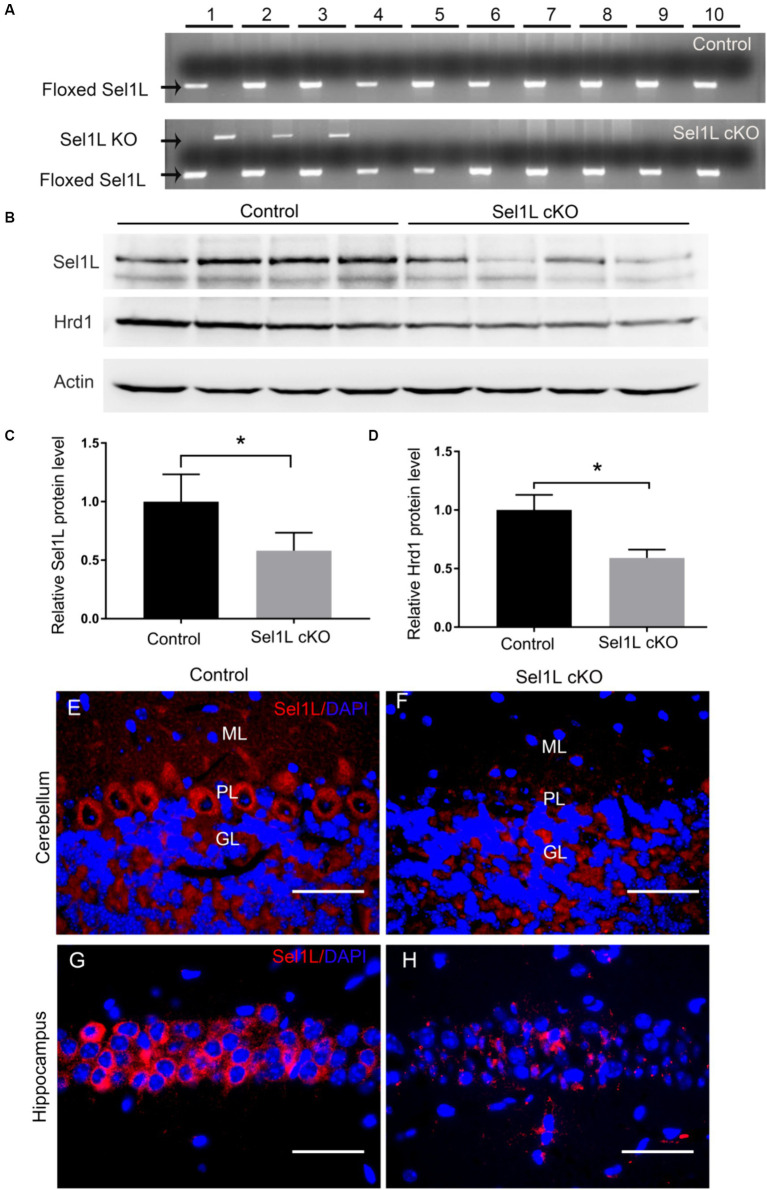
Sel1L was deleted selectively in neurons in the CNS of Sel1L cKO mice. **(A)** PCR analysis showed that the floxed Sel1L allele was present in all tissues of Sel1L cKO mice and control mice, but the Sel1L knockout (KO) allele was only present in the CNS of Sel1L cKO mice. 1, cerebrum; 2, cerebellum; 3, spinal cord; 4, optic nerve; 5, sciatic nerve; 6, heart; 7, liver; 8, spleen; 9, lung; 10, kidney. *N* = 4 animals. **(B,C)** Western blot analysis showed decreased Sel1L protein level in the brain of Sel1L cKO mice compared to control mice. *N* = 4 animals. **(B,D)** Western blot analysis showed decreased Hrd1 protein level in the brain of Sel1L cKO mice compared to control mice. *N* = 4 animals. **(E,F)** Sel1L immunostaining showed that the immunoreactivity of Sel1L was detectable in Purkinje neurons of control mice, but became undetectable in Purkinje neurons of Sel1L cKO mice. *N* = 4 animals. **(G,H)** Sel1L immunostaining showed that the immunoreactivity of Sel1L was detectable in hippocampal neurons of control mice, but became undetectable in hippocampal neurons of Sel1L cKO mice. *N* = 4 animals. Error bars represent SD. *p < 0.05. Scale bars: 50 μm.

To explore the role of ERAD in neurons, we generated a mouse model that allows for inactivation of Sel1L specifically in neurons in the CNS of adult mice. *Sel1L^loxP^* mice were crossed with *Thy1/CreER^T2^* mice, and the resulting progeny were further crossed with *Sel1L^loxP^* mice to obtain *Sel1L^loxP/loxP^*; *Thy1/CreER^T2^* mice and *Sel1L^loxP/loxP^* mice. To induce Sel1L deletion specifically in neurons of adult mice, *Sel1L^loxP/loxP^; Thy1/CreER^T2^* mice (Sel1L cKO mice) and *Sel1L^loxP/loxP^* mice (control mice) were given intraperitoneal injections of tamoxifen daily for 10 days starting at the age of 8 weeks. Brain tissues were prepared from Sel1L cKO mice and control mice at post-injection of tamoxifen day 7 (PID 7). PCR analysis of genomic DNA confirmed the deletion of exon 6 of the *Sel1L* gene selectively in the cerebrum, cerebellum and spinal cord of Sel1L cKO mice, but not in other organs of Sel1L cKO mice or in any organs of control mice (Figure 1A).

The Sel1L-Hrd1 complex is the best-characterized ERAD machinery (Gao Y. et al., 2023; Ji et al., 2023). It is known that Hrd1 is an ER-resident E3 ligase, its transmembrane regions form a retrotranslocation channel to export the ER proteins and its cytoplasmic RING finger domain ubiquitinizes the proteins (Baldridge and Rapoport, 2016; Wu and Rapoport, 2018). Sel1L, an ER transmembrane protein, interacts with Hrd1 and blocks Hrd1 autoubiquitination (Sun et al., 2014; Gao Y. et al., 2023; Ji et al., 2023). A large number of studies have demonstrated that Sel1L is essential for Hrd1 stability and the ERAD activity of the SelL-Hrd1 complex (Sun et al., 2014; Wu et al., 2021; Gao Y. et al., 2023; Ji et al., 2023; Wu and Lin, 2023). Western blot analysis showed that the protein levels of Sel1L and Hrd1 were significantly decreased in the brain of Sel1L cKO mice compared to control mice (Figures 1B–D). Sel1L immunostaining showed that the immunoreactivity of Sel1L was detectable in the majority of Purkinje neurons and hippocampus neurons of control mice, but became undetectable in these neurons of Sel1L cKO mice (Figures 1E–H). These data suggest that tamoxifen treatment induces Sel1L deletion specifically in neurons and results in elimination of Hrd1 protein and impairment of ERAD in neurons in the CNS of adult Sel1L cKO mice.

**Figure 2:**
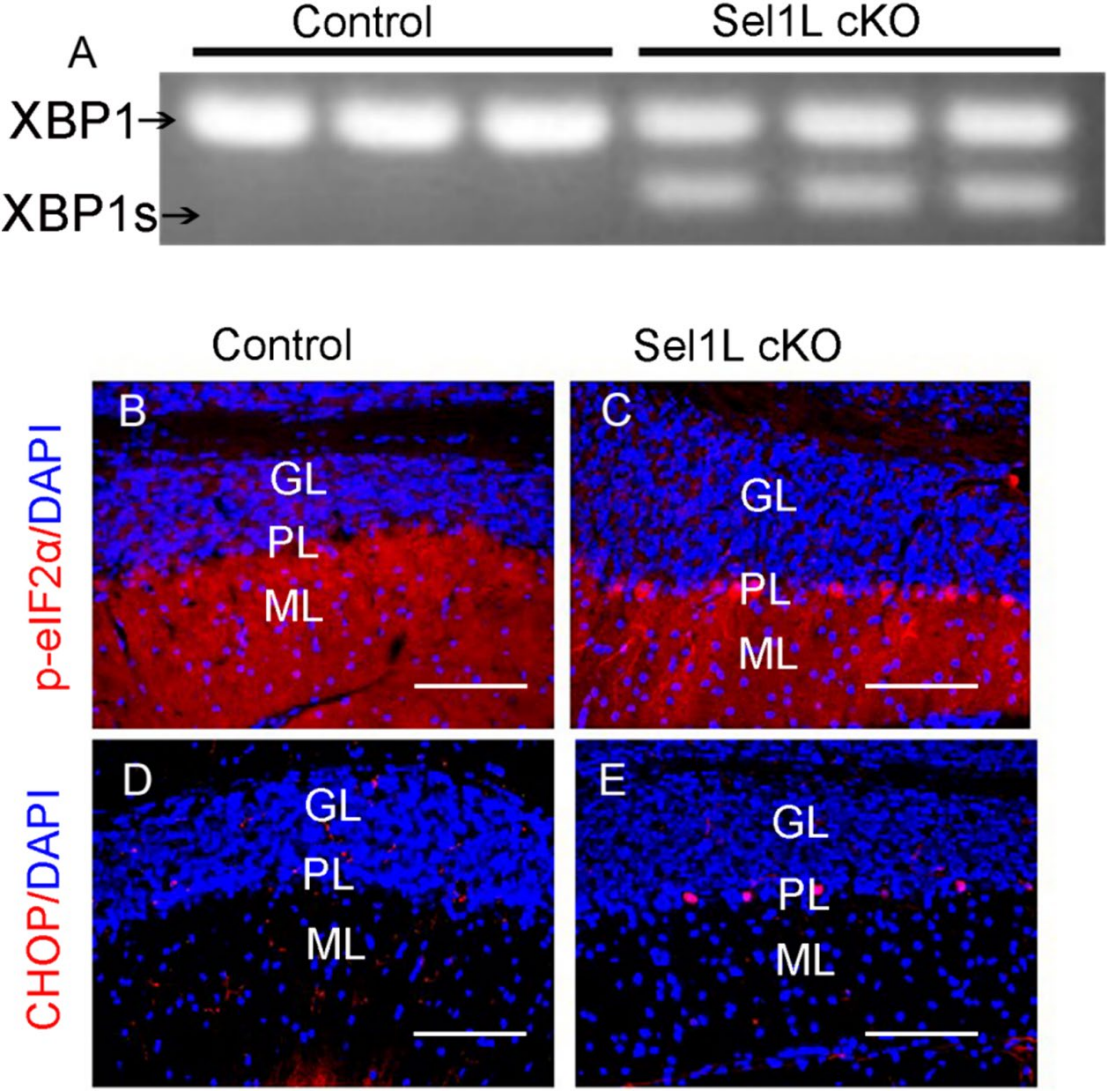
Neuron-specific Sel1L inactivation in adult mice resulted in activation of the UPR in neurons in the CNS. **(A)** PCR analysis showed the XBP1s became detectable in the brain of Sel1L cKO mice. *N* = 3 animals. **(B,C)** p-eIF2α immunostaining showed that the immunoreactivity of p-eIF2α was noticeably increased in Purikinje neurons of Sel1L cKO mice compared to control mice. *N* = 4 animals. **(D,E)** CHOP immunostaining showed that the immunoreactivity of CHOP was noticeably increased in Purkinje neurons of Sel1L cKO mice compared to control mice. *N* = 4 animals. ML, Molecular layer; PL, Purkinje cell layer; GL, Granular layer. Scale bars: 100 μm.

Data indicate that impairment of ERAD induced by Sel1L deficiency leads to ER stress and activation of the UPR, including activation of the IRE1 and PERK branches, in cells (Sun et al., 2014; Wu et al., 2020, 2021; Gao Y. et al., 2023). IRE1 activation upregulates the expression of genes that enhance protein folding and protein degradation by splicing X-Box Binding Protein 1 (XBP1) mRNA (Clayton and Popko, 2016; Taylor and Hetz, 2020; Wiseman et al., 2022; Wu and Lin, 2024). PERK activation inhibits global protein translation but stimulates the expression of numerous stress-responsive genes (including CHOP) by phosphorylating eIF2α (Clayton and Popko, 2016; Taylor and Hetz, 2020; Wiseman et al., 2022; Wu and Lin, 2024). To determine whether impaired ERAD leads to disruption of ER homeostasis and activation of the UPR, we performed PCR analysis to determine XBP1 mRNA splicing. As expected, spliced XBP1 (XBP1s) mRNA was undetectable in the brain of control mice at PID 7. Importantly, XBP1s mRNA was detectable in the brain of Sel1L cKO mice (Figure 2A). Moreover, phosphorylated eIF2α (p-eIF2α) immunostaining showed that immunoreactivity of p-eIF2α was noticeably increased in Purkinje neurons of Sel1L cKO mice, as compared to control mice (Figures 2B,C). Similarly, CHOP immnostaining showed that immunoreactivity of CHOP was noticeably increased in Purkinje neurons of Sel1L cKO mice, as compared to control mice (Figures 2D,E). Collectively, these data suggest that impairment of the ERAD activity of the Sel1L-Hrd1 complex disrupts ER homeostasis and leads to activation of the UPR in neurons.

**Figure 3:**
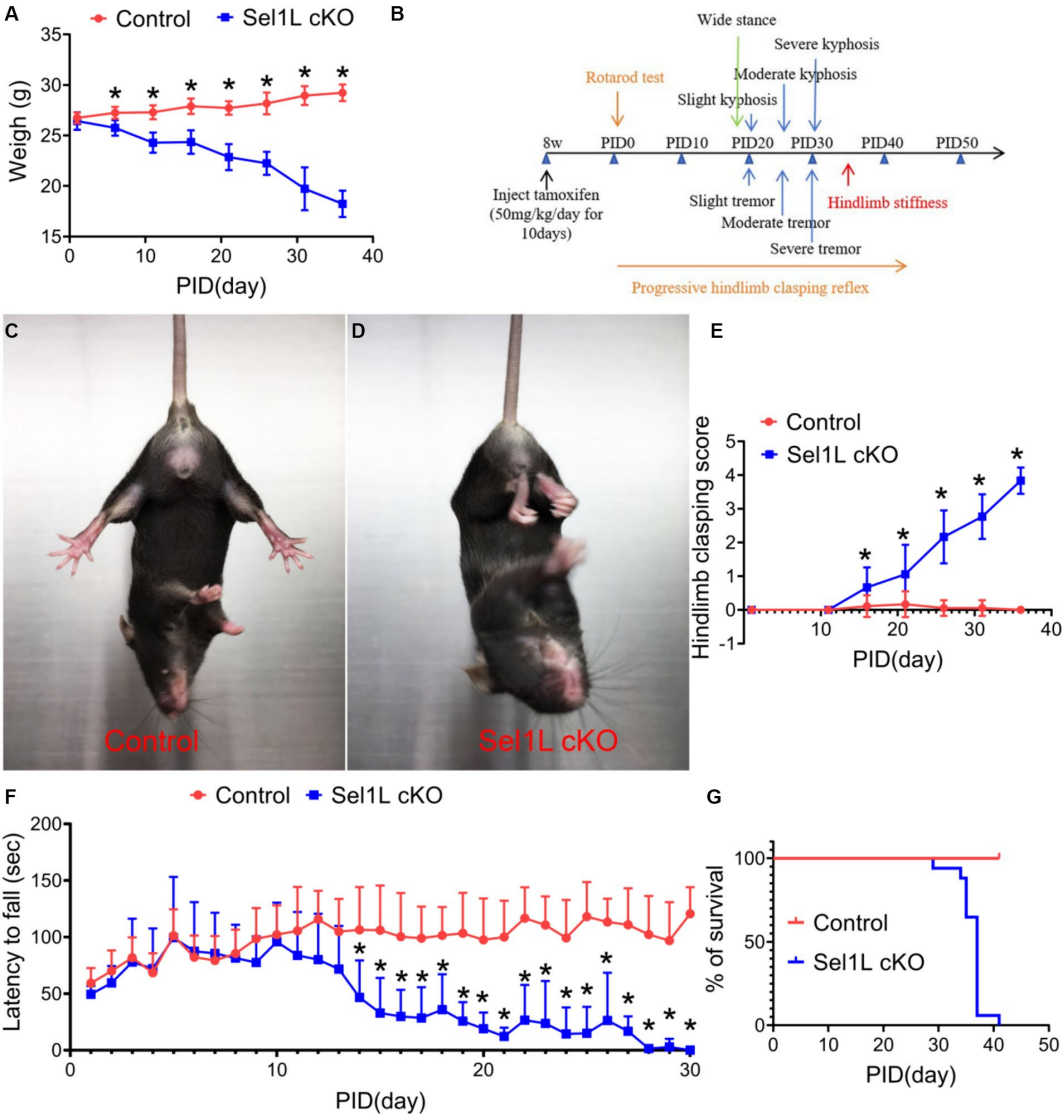
Neuron-specific Sel1L inactivation in adult mice led to weight loss, motor dysfunction, and death. **(A)** Sel1L cKO mice displayed progressive weight loss, as compared to control mice. *N* = 8–12 animals. **(B)** Timeline of Sel1L cKO mice developing progressive tremor, wide stance, kyphosis and hindlimb stiffness after tamoxifen injection. *N* = 8–12 animals. **(C–E)** Hindlimb clasping score showed a significant increase of hindlimb clasping score in Sel1L cKO mice compared to control mice. *N* = 8–12 animals. **(F)** Rotarod test showed that the latency to fall of Sel1L cKO mice was significantly decreased compared to control mice. *N* = 8–12 animals. **(G)** Survival curve showed that all Sel1L cKO mice died around PID 40. *N* = 8–12 animals. Error bars represent SD. *p < 0.05.

We monitored the Sel1L cKO mice daily. Sel1L cKO mice showed progressive weight loss starting at PID 5 as compared to control mice (Figure 3A). Sel1L cKO mice also displayed progressive tremor, wide stance, hunched back, hindlimb stiffness starting around PID 20 (Figure 3B). The degree of clasping in the hindlimb clasping test is used to assess severity of motor dysfunction in neurodegeneration (Liu et al., 2023). Control mice showed a normal extension reflex in the hindlimbs and used body torsion to try to grab their tails when suspended in the air; however, Sel1L cKO mic exhibited significantly increased and progressive hindlimb clasping severity compared to control mice starting at PID 15 (Figures 3C–E), suggesting progressive hindlimb clasping reflex in Sel1L cKO mice. Moreover, accelerating rotarod test showed that the latency to fall of Sel1L cKO mice was significantly decreased compared to control mice starting at PID 14 (Figure 3F), indicating progressive impairment of motor activity in Sel1L cKO mice. Since weight loss occurred in Sel1L cKO mice prior to motor dysfunction, the contribution of motor dysfunction to weight loss in Sel1L cKO mice can be ruled out. Notably, all Sel1L cKO mice succumbed to death around PID 40 (Figure 3G), demonstrating that the ERAD activity of the Sel1L-Hrd1 complex in neurons is required for animal survival under normal conditions. Collectively, these data demonstrate that neuron-specific Sel1L inactivation in adult mice led to weight loss, motor dysfunction, and death.

### 3.2 Neuron-specific Sel1L inactivation led to global atrophy of the brain in adult mice

**Figure 4:**
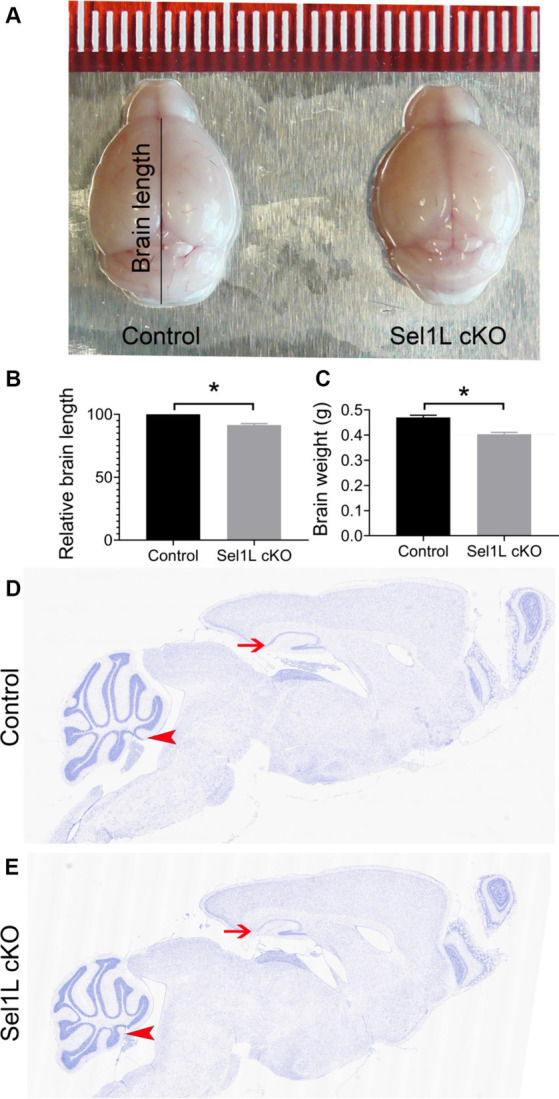
Neuron-specific Sel1L inactivation led to global atrophy of the brain in adult mice. **(A)** Representative brain images of Sel1L cKO mice and control mice. *N* = 3 animals. **(B)** Brain length of Sel1L cKO mice was significantly decreased as compared to control mice. *N* = 3 animals. **(C)** Brain weight was significantly decreased in Sel1L cKO mice compared to control mice. *N* = 5–6 animals. **(D,E)** Representative Nissl staining images of whole brain from Sel1L cKO mice and control mice. *N* = 4 animals. Atrophy of the hippocampus (arrows) was noticeable in the Sel1L cKO mice compared to control mice. Atrophy of the cerebellum (arrowheads) was noticeable in the Sel1L cKO mice compared to control mice.

The clinical phenotypes displayed by Sel1L cKO mice imply neurodegeneration in the CNS. Brain tissues were prepared from Sel1L cKO mice and control mice at PID 37. We found that the size of the brain of Sel1L cKO mice was noticeably reduced compared to control mice (Figures 4A,B). Similarly, the weight of the brain of Sel1L cKO mice was significantly reduced compared to control mice (Figure 4C). Moreover, whole brain scanning images of Nissl staining revealed prominent atrophy of the cerebellum and hippocampus and modest atrophy of other brain regions in Sel1L cKO mice compared to control mice (Figures 4D,E). These data suggest that Sel1L inactivation in neurons leads to global atrophy of the brain, particularly atrophy of the cerebellum and hippocampus, in adult mice.

### 3.3 Neuron-specific Sel1L inactivation led to degeneration of Purkinje neurons in adult mice

The clinical phenotypes exhibited by Sel1L cKO mice partially replicate cerebellar ataxia, including progressive tremor, wide stance, hunched back, hindlimb stiffness, progressive hindlimb clasping reflex, and decreased latency to fall in the rotarod test. Whole brain scanning images of Nissl staining also revealed prominent atrophy of the cerebellum in Sel1L cKO mice. Purkinje neurons, a unique type of neuron-specific to the cerebellar cortex, are characterized by very extensive and elaborate dendritic branches and play a fundamental role in controlling motor movement (Fujishima et al., 2018; Hirano, 2018). It is well documented that degeneration of Purkinje neurons leads to cerebellar ataxia. Therefore, we determined the effects of Sel1L inactivation on Purkinje neurons.

**Figure 5:**
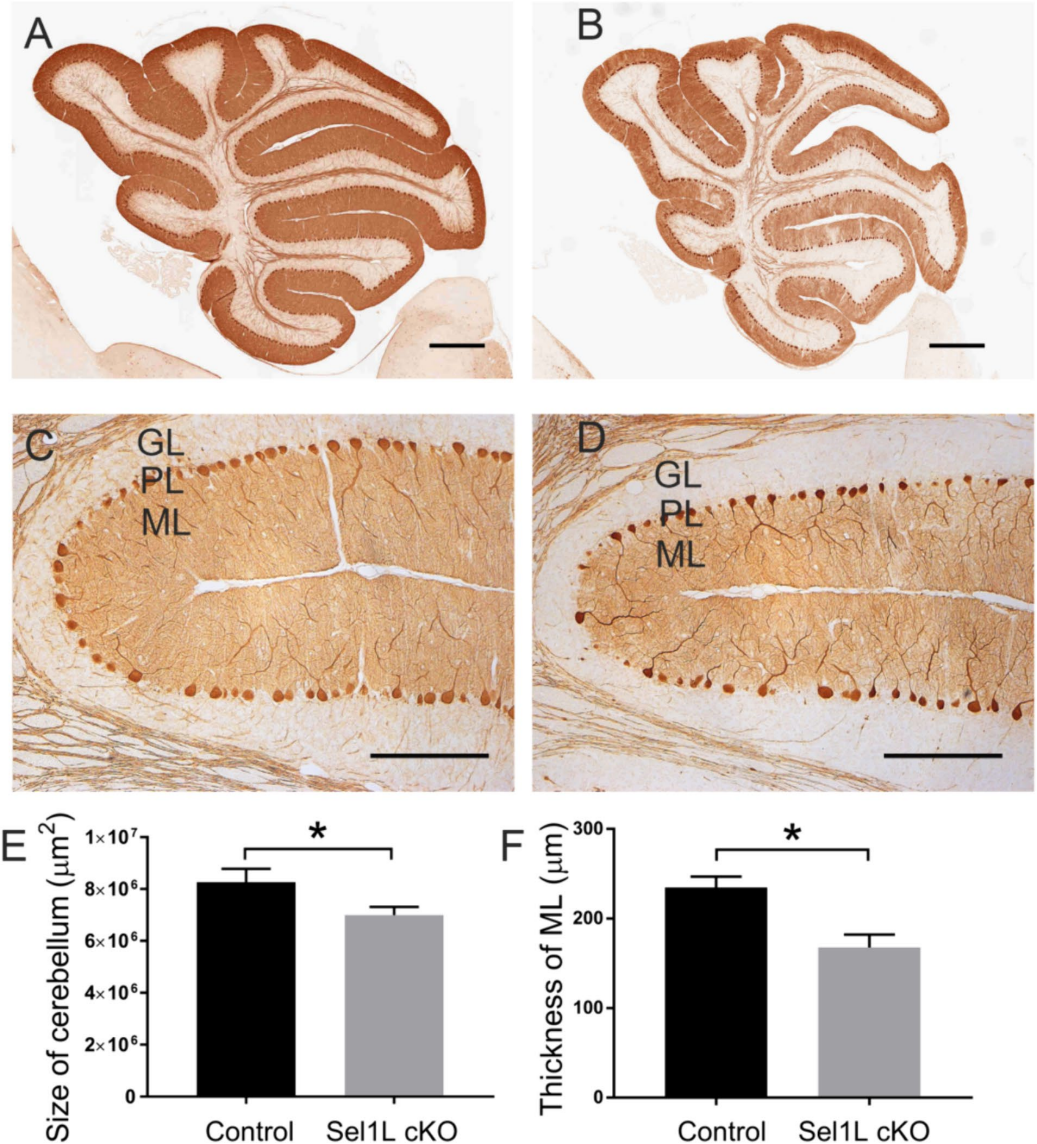
Neuron-specific Sel1L inactivation led to cerebellum atrophy in adult mice. **(A,B,E)** Calbindin IHC showed the reduced size of the cerebellum in Sel1L cKO mice compared to control mice. *N* = 4 animals. **(C,D,F)** Calbindin IHC showed the reduced thickness of the molecular layer in the cerebellum of Sel1L cKO mice compared to control mice. *N* = 4 animals. Error bars represent SD. *p < 0.05. Scale bars: 100 μm.

**Figure 6:**
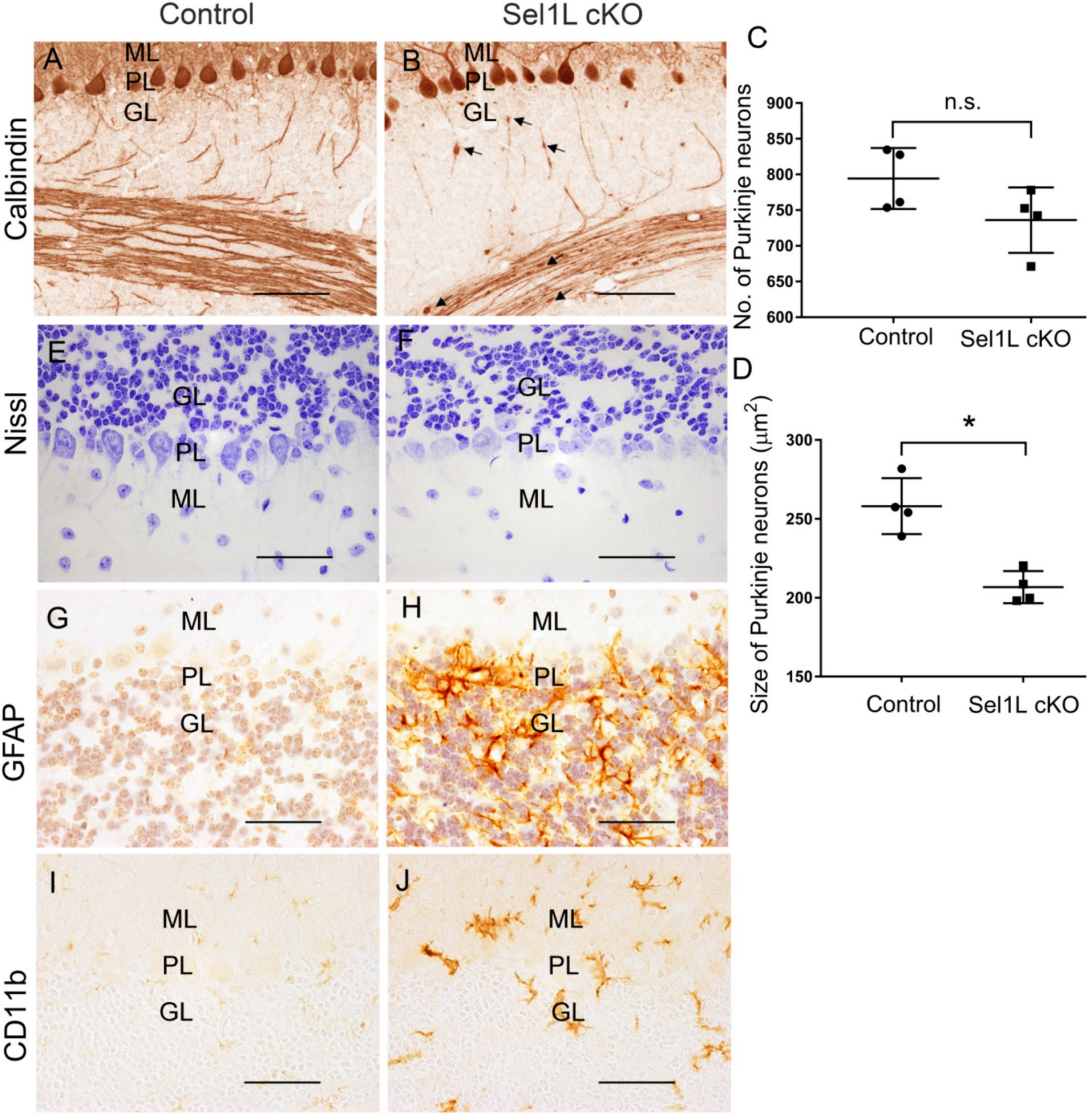
Neuron-specific Sel1L inactivation led to degeneration of Purkinje neurons and gliosis in cerebellum of adult mice. **(A–D)** Calbindin IHC showed that the number of Purkinje neurons was moderately but not significantly reduced in the cerebellum of Sel1L cKO mice compared to control mice, and the soma size of Purkinje neurons was significantly decreased in the cerebellum of Sel1L cKO mice compared to control mice. Calbindin IHC also revealed swollen axons (arrows) of Purkinje neurons in the granular layer of the cerebellum in Sel1L cKO mice. *N* = 4 animals. **(E,F)** Nissl staining revealed dramatic decrease of cytoplasmic basophilic Nissl substance in Purkinje neurons of Sel1L cKO mice compared to control mice. *N* = 4 animals. **(G,H)** GFAP IHC showed astrocyte activation in the cerebellum of Sel1L cKO mice compared to control mice. *N* = 4 animals. **(I,J)** CD11b IHC showed microglia activation in the cerebellum of Sel1L cKO mice compared to control mice. *N* = 4 animals. Error bars represent SD. *p < 0.05. Scale bars: 50 μm.

Calbindin (a maker for Purkinje neurons) IHC showed that the size of the cerebellum was significantly reduced in Sel1L cKO mice compared to control mice at PID 37 (Figures 5A,B,E). The thickness of the molecular layer in the cerebellum of Sel1L cKO mice was also significantly reduced compared to control mice at PID 37 (Figures 5C,D,F). While calbindin IHC showed that the number of Purkinje neurons was moderately but not significantly reduced in the cerebellum of Sel1L cKO mice and control mice at PID 37 (Figures 6A–C), quantitative analysis showed that the soma size of Purkinje neurons was significantly decreased in Sel1L cKO mice compared to control mice at PID 37 (Figures 6A,B,D), suggesting atrophy of Purkinje neurons in the cerebellum of Sel1L cKO mice. Calbindin IHC also revealed swollen axons of Purkinje neurons in the granular layer of the cerebellum in Sel1L cKO mice (Figures 6A,B). Moreover, Nissl staining revealed dramatic decrease of cytoplasmic basophilic Nissl substance in Purkinje neurons of Sel1L cKO mice compared to that of control mice (Figures 6E,F). Additionally, it is known that astrocytes and microglia are activated in response to degeneration of Purkinje neurons (Vandenbark et al., 2021; Rosa et al., 2022; Gao C. et al., 2023). GFAP (a marker of astrocytes) IHC showed marked astrocyte activation in the cerebellum of Sel1L cKO mice compared to control mice at PID37 (Figures 6G,H). CD11b (a marker of microglia) staining showed marked microglia activation in the cerebellum of Sel1L cKO mice compared to control mice at PID37 (Figures 6I,J). Taken together, these results suggest that Sel1L inactivation in neurons leads to degeneration of Purkinje neurons and results in the clinical phenotypes of cerebellar ataxia in adult Sel1L cKO mice.

### 3.4 Neuron-specific Sel1L inactivation led to hippocampal degeneration in adult mice

**Figure 7:**
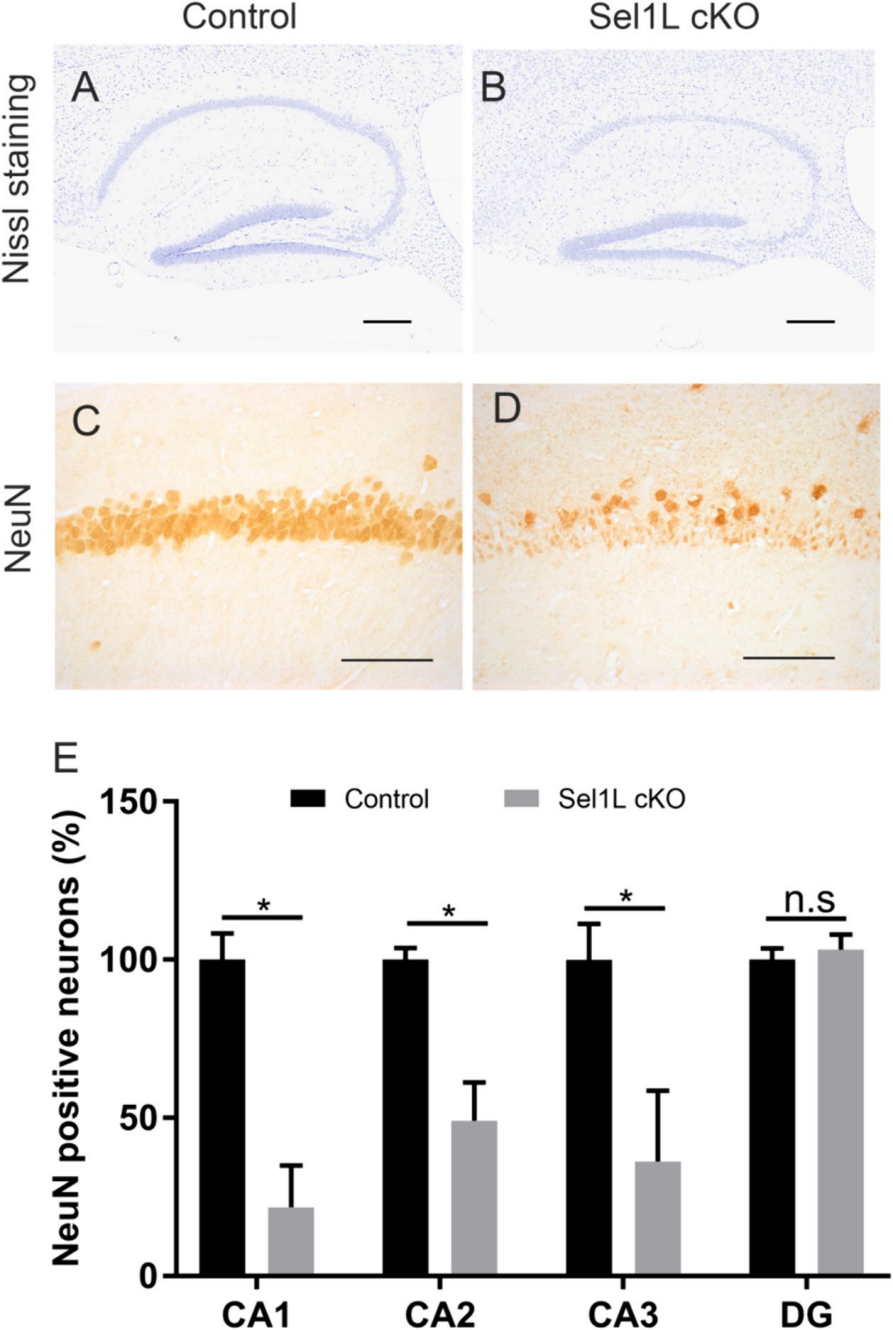
Neuron-specific Sel1L inactivation led to hippocampal degeneration in adult mice. **(A,B)** Nissl staining showed hippocampal neuron loss in Sel1L cKO mice compared to control mice. *N* = 4 animals. **(C–E)** NeuN IHC showed significant neuron loss in the CA1 layer, CA2 layer and CA3 layer in the hippocampus of Sel1L cKO mice compared to control mice. *N* = 4 animals. Error bars represent SD. *p < 0.05. Scale bars: **(A,B)** 100 μm; **(C,D)** 50 μm.

**Figure 8:**
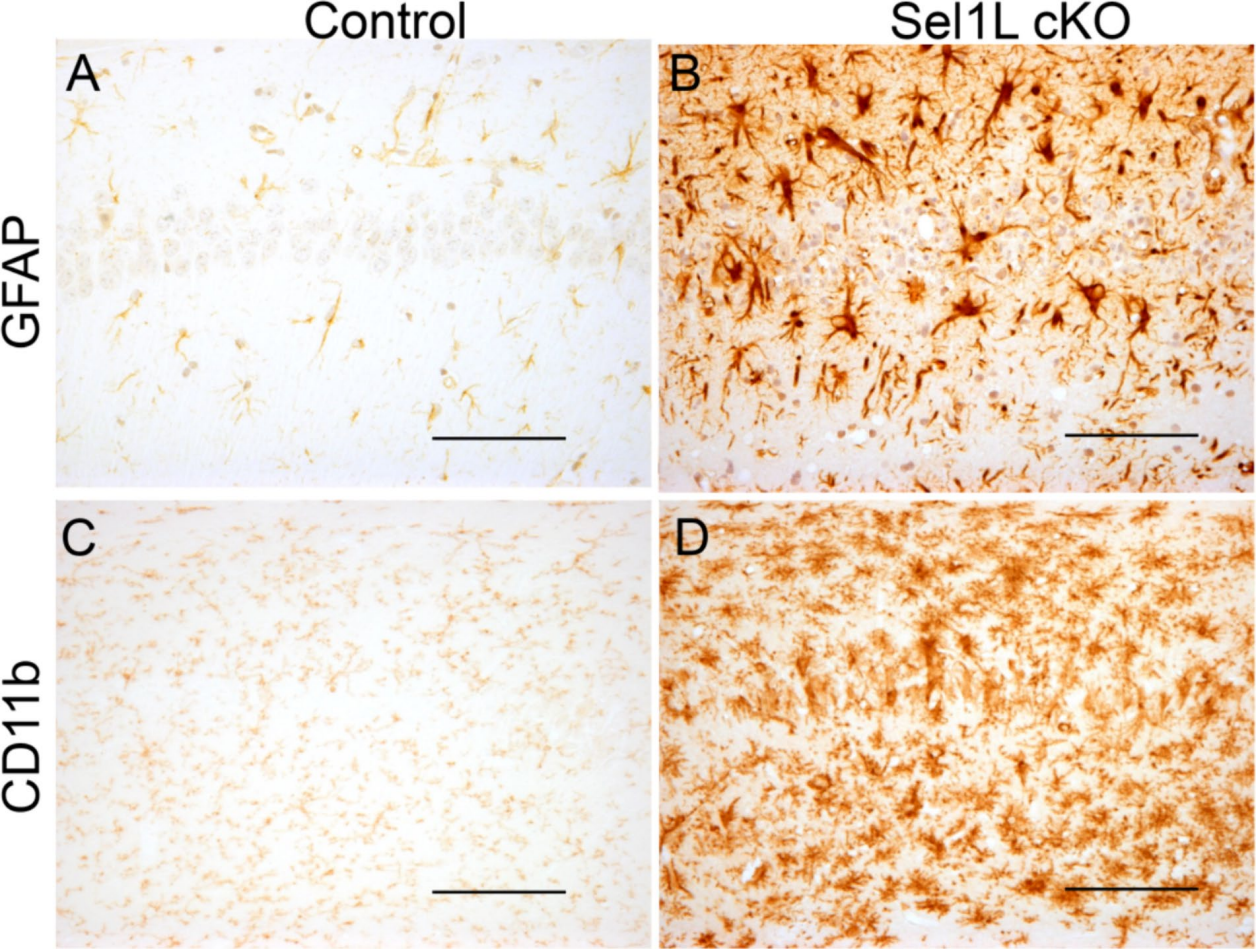
Neuron-specific Sel1L inactivation led to gliosis in the hippocampus of adult mice. **(A,B)** GFAP IHC showed astrocyte activation in the hippocampus of Sel1L cKO mice compared to control mice. *N* = 4 animals. **(C,D)** CD11b IHC showed microglia activation in the hippocampus of Sel1L cKO mice compared to control. *N* = 4 animals. Scale bars: **(A–D)** 50 μm.

Our previous study has shown that disruption of ER homeostasis in neurons can lead to hippocampal degeneration in adult mice (Liu et al., 2023). We showed here that Sel1L inactivation in neurons caused disruption of ER homeostasis. Whole brain scanning images of Nissl staining also revealed prominent atrophy of the hippocampus in adult Sel1L cKO mice. Unfortunately, Sel1L cKO mice exhibited severe motor dysfunction; we could not perform behavioral tests, such as the Barnes maze test, to evaluate spatial learning and memory in these mice. High magnification images of Nissl staining revealed severe hippocampal neuron loss in Sel1L cKO mice compared to control mice at PID 37 (Figures 7A,B). Quantitative NeuN IHC showed dramatic neuron loss in the CA1 layer and severe neuron loss in the CA2 layer and CA3 layer in Sel1L cKO mice compared to control mice at PID37 (Figures 7C–E). Nevertheless, the number of neurons in the dentate gyrus (DG) of Sel1L cKO mice was comparable to control mice (Figures 7C–E). Moreover, it is well documented that astrocytes and microglia are activated in response to degeneration of hippocampal neurons (Liu et al., 2023). Accordingly, GFAP IHC revealed marked activation of astrocyte in the hippocampus of Sel1L cKO mice compared to control mice at PID 37 (Figures 8A,B). CD11b IHC revealed marked activation of microglia in the hippocampus of Sel1L cKO mice compared to control mice at PID37 (Figures 8C,D). Collectively, these results suggest that Sel1L inactivation in neurons results in degeneration of hippocampal neurons in adult mice.

## 4 Discussion

ERAD is responsible for recognition and degradation of unfolded or misfolded proteins in the ER (Sun et al., 2014; Qi et al., 2017; Wu et al., 2021; Wu and Lin, 2023). The Sel1L-Hrd1 complex is the best-characterized ERAD component. Numerous studies have established that Sel1L is vital for maintaining Hrd1 stability and the overall ERAD activity of the Sel1L-Hrd1 complex (Sun et al., 2014; Wu et al., 2020, 2021; Ji et al., 2023). Sel1L-Hrd1 ERAD ablation in AVP (argininevasopressin) neuron or POMC (pro-opiomelanocortin) neurons impacts the whole-body metabolism (Shi et al., 2017; Kim et al., 2018). Moreover, a recent report showed that Sel1L contributes to neuron differentiation and maturation, including synaptogenesis (Saito et al., 2023). Thus, to investigate the role of ERAD in preserving ER homeostasis and the function and viability of neurons, we generated a conditional knockout mice model with Sel1L inactivation specifically in neurons in the CNS (Sel1L cKO mice). As expected, we observed that disrupting ERAD through the elimination of the Sel1L-Hrd1 complex caused disruption of ER homeostasis and activation of the UPR in neurons in the CNS of adult mice. Interestingly, this impairment in ERAD led to global brain atrophy and neurodegeneration, particularly impacting Purkinje neurons and hippocampus neurons, resulting in weight loss, motor dysfunction, and death of mice. These findings underscore the significance of ERAD in regulating ER homeostasis in neurons and in sustaining their function and viability under physiological conditions.

In a genome-wide association study involving 31 dogs, the gene associated with ataxia was located within a 1.5 Mb region on canine chromosome 8, with Sel1L identified as the most likely candidate gene for mutation (Kyostila et al., 2012). This mutation in Sel1L has been linked to the loss of Purkinje neurons and the development of progressive early-onset cerebellar ataxia in dogs (Kyostila et al., 2012). Additionally, recent human studies have found that mutations in Sel1L or Hrd1 are associated with developmental delay and locomotor dysfunction, including ataxia (Wang et al., 2024). Accordingly, in our study, the dominant phenotypes of Sel1L cKO mice were ataxia and tremor, suggesting that Sel1L inactivation in neurons is a significant cause of cerebellar ataxia. We found that Sel1L inactivation in neurons moderately but not significantly reduced the number of Purkinje neurons in the cerebellum of adult mice. Importantly, we found that Sel1L inactivation in Purkinje neurons led to reduced soma size, swollen axons, and decrease of cytoplasmic Nissl substance, and results in the reduced thickness of the molecular layer in the cerebellum. Moreover, there was noticeable activation of microglia and astrocytes in the cerebellum of Sel1L cKO mice. These findings demonstrate that Sel1L inactivation in neurons caused degeneration of Purkinje neurons in the cerebellum of adult mice. However, due to the rapid demise observed in Sel1L cKO mice, it is possible that their survival time is insufficient to permit the progression of Purkinje neuron degeneration to death. Taken together, these data suggest that the impaired ERAD activity of Sel1L-Hrd1 complex disrupts ER homeostasis in Purkinje neurons and results in their degeneration in adult mice, and imply the essential role of ERAD in maintaining the function and viability of Purkinje neurons in adult mice under normal and disease conditions.

On the other hand, ERAD deficiency is associated with the development of β-amyloid pathology, hippocampal neuronal death, and aggravated memory impairments in the mouse model of Alzheimer’s disease (Zhu et al., 2017). Data indicate that disruption of ER homeostasis and ER stress is a major contributor to the dysfunction and death of hippocampal neurons in neurodegenerative diseases (Zheng et al., 2019; Ajoolabady et al., 2022). While deficiency of individual branches of the UPR (either PERK, ATF6α, or IRE1) in neurons does not cause disruption of ER homeostasis and ER stress or lead to neurodegeneration in adult animals (Ma et al., 2013; Yoshikawa et al., 2015; Duran-Aniotz et al., 2017; Qi et al., 2017; Stone et al., 2019)_,_ double deletion of PERK and ATF6α in neurons leads to disruption of ER homeostasis and results in neurodegeneration in the CNS of adult mice, especially impairment of spatial memory and hippocampal degeneration. Interestingly, we showed here that Sel1L inactivation specifically in neurons caused disruption of ER hemostasis and led to dramatic neuron loss in the CA1 layer, severe neuron loss in the CA2 layer and CA3 layer, but minimal neuron loss in the DG in the hippocampus of adult mice, which are similar to the mice with double deletion of PERK and ATF6α in neurons. Collectively, these data suggest that the impaired ERAD activity of the Sel1L-Hrd1 complex disrupts ER homeostasis in hippocampal neurons and results in their degeneration in adult mice, and imply the essential role of ERAD in maintaining the function and viability of hippocampal neurons in adult mice under normal and disease conditions.

In summary, our study represents an initial demonstration of the critical role of ERAD in maintaining ER homeostasis and the function and viability of neurons in the CNS, particularly Purkinje neurons and hippocampus neurons. Our previous studies have shown that Sel1L inactivation in myelinating cells, oligodendrocytes in the CNS and Schwann cells in the PNS, leads to their dysfunction and death through activation of the UPR (Wu et al., 2020, 2021; Wu and Lin, 2023). We showed here that Sel1L inactivation in neurons caused activation of the UPR and neurodegeneration in adult mice. However, the role of the UPR in neurodegeneration resulting from Sel1L inactivation in neurons remains unclear and requires further investigation.

## Data availability statement

The original contributions presented in the study are included in the article/supplementary material, further inquiries can be directed to the corresponding author.

## Ethics statement

The animal study was approved by Institutional Animal Care and Use Committee of the University of Minnesota. The study was conducted in accordance with the local legislation and institutional requirements.

## Author contributions

SW: Data curation, Writing – original draft, Writing – review & editing. PL: Data curation, Investigation, Writing – review & editing. MC: Writing – review & editing. WL: Supervision, Writing – review & editing.
